# Computational Methods for Mapping, Assembly and Quantification for Coding and Non-coding Transcripts

**DOI:** 10.1016/j.csbj.2019.04.012

**Published:** 2019-05-07

**Authors:** Isaac A. Babarinde, Yuhao Li, Andrew P. Hutchins

**Affiliations:** Department of Biology, Southern University of Science and Technology, 1088 Xueyuan Lu, Shenzhen, China

**Keywords:** RNA-Seq, Transcript, Genome, Transposable element, Long non-coding RNA

## Abstract

The measurement of gene expression has long provided significant insight into biological functions. The development of high-throughput short-read sequencing technology has revealed transcriptional complexity at an unprecedented scale, and informed almost all areas of biology. However, as researchers have sought to gather more insights from the data, these new technologies have also increased the computational analysis burden. In this review, we describe typical computational pipelines for RNA-Seq analysis and discuss their strengths and weaknesses for the assembly, quantification and analysis of coding and non-coding RNAs. We also discuss the assembly of transposable elements into transcripts, and the difficulty these repetitive elements pose. In summary, RNA-Seq is a powerful technology that is likely to remain a key asset in the biologist's toolkit.

## Graphical Abstract

**Figure:**
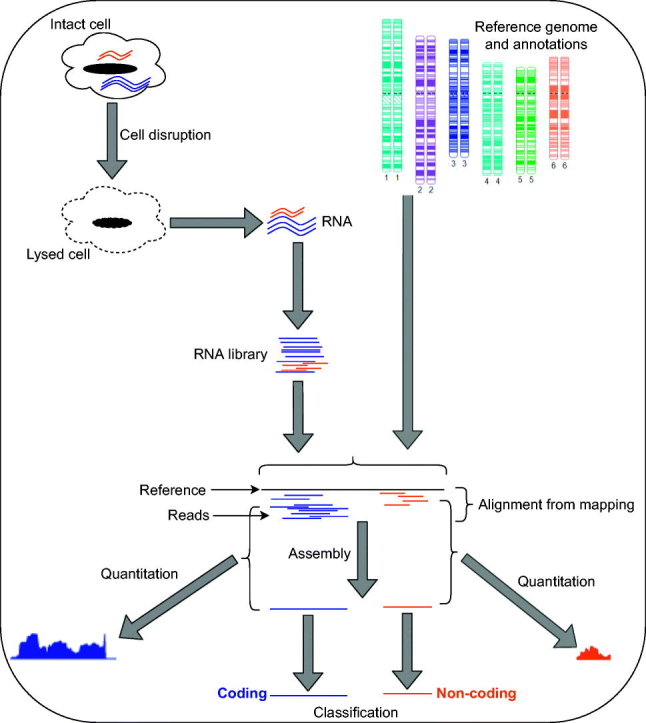

## 1 Introduction

**Fig. 1:**
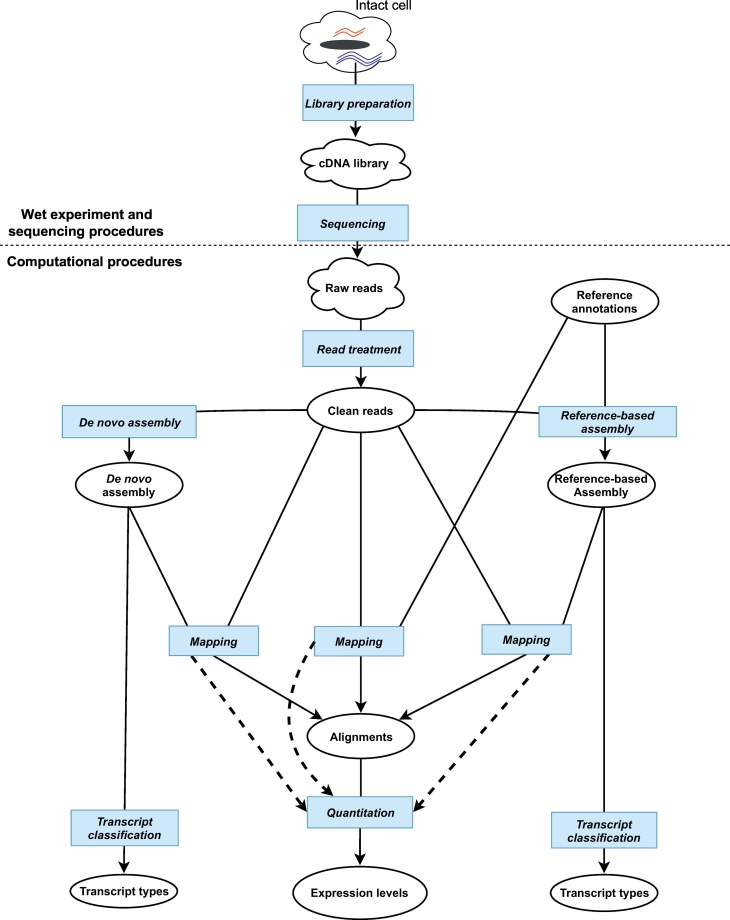
Typical decision lines in coding and non-coding transcript assembly. The upper part summarizes wet experimental procedures required to produce RNA-Seq reads. The lower part highlights the computational analyses and decision lines. Transcript assembly starts with the evaluation of read quality, and can proceed with or without reference annotations. Blue square boxes denote decision points on tools to use, and arrows denote strategic considerations in how to analyze the RNA-Seq data. Dotted lines indicate optional pathways. (For interpretation of the references to colour in this figure legend, the reader is referred to the web version of this article.)

The relationship between gene expression dynamics and biological function has long been explored [[1], [2], [3]]. Whilst it is clear that measuring gene expression cannot capture all of the cell's information content, the ease of manipulation of nucleic acids has led to the widespread adoption of gene expression measures to many domains of biology. Recent innovations, first in microarray [4,5], and then in sequencing technologies [6,7], substantially drove down the cost and increased the throughput of measuring RNA gene expression, so much so, that a search for the keywords “differential gene expression” on NCBI PubMed, returned 68,519 hits. RNA sequencing (RNA-Seq), has become a dominant technique in measuring gene expression levels [6,[8], [9], [10], [11], [12]]. Indeed, measuring gene expression through RNA-Seq technology has become near ubiquitous in biomedical research and studies now often sequence hundreds of samples [[13], [14], [15]]. However, there are many known and unknown biases in the quantification of RNAs, and efforts have been made to mitigate these effects [[16], [17], [18], [19], [20], [21]]. In many cases, the choice of analysis strategy that the researcher wishes to perform determines which of these biases are critical, and which can be safely ignored. Despite technological innovations, many RNA-Seq gene abundance estimate techniques require the disruption of tissues and cells, followed by the extraction of RNA, fragmentation, amplification and/or size-selection. Although newer sequencing technologies aim to dispense with amplification by PCR (polymerase chain reaction), it is currently used in most common RNA-Seq protocols, and PCR is well known to be biased [16,21,22]. These and other processes usually complicate RNA abundance estimates of gene expression by contributing unseen biases in the data. In addition, the expression levels of certain transcripts in the same sample are heterogeneous, leading to stochasticity in the estimates [23,24]. Indeed, there is a number of confounding issues at almost all stages of the analysis of gene expression, and a number of bioinformatics tools have been developed to handle specific steps and biases in the process of capturing the expression levels. Here, starting with the assessment and treatment of sequence reads, we review commonly used bioinformatics methods, available tools and strategic considerations for the assembly and quantification of gene expression (Fig. 1), including a discussion of the case of assembling transcripts that contain repetitive transposable elements, which pose their own special challenges. We conclude by giving insights into the factors to consider in deciding which bioinformatics tools or pipelines to use.

## 2 Sequencing Platform Technologies and Pipelines

The most common form of high throughput sequencing is ‘short-read’ sequencing, where the read length can range up to 300 bp in length. This approach in transcriptome analyses is commonly referred to as ‘RNA-Seq’. In this approach, RNA is extracted, fragmented, converted to cDNA, amplified and sequenced (Fig. 1). The processing of RNA to a form ready for sequencing [25] is known as library preparation, and is an important initial step in RNA-Seq [26,27], that is constantly being improved [10,[28], [29], [30]]. If the RNA is collected from tissue, the first step is tissue rupture, followed by cell lysis, purification and reverse transcription to get cDNA [25]. In the case of short RNAs such as miRNAs, the short RNA molecules are size-selected using gel electrophoresis [31]. Longer transcripts can be selected by using oligodT or ribosomal RNA depletion and then fragmented before reverse transcription [25,27,29,30]. RNA-Seq library is thereafter sequenced to get “reads”. Gene expression estimates are then made by counting the number of reads that align to each transcript, to arrive at an estimate of the quantity of RNA in the original sample.

The underlying sequencing technology can be either single-ended (the fragment is sequenced from only one end) or paired-end (the fragments is sequenced from both ends). In general, for RNA-Seq, it is desirable to have the longest possible paired-end reads, to achieve the best mapping coverage and the highest chance of observing splice junctions. However, the transcript type of interest will determine the read length of choice. For example, in the study of microRNAs and other very short RNAs, sequencing lengths must be necessarily small, as the RNAs themselves are short. Conversely, for coding and long non-coding RNAs, in general, the longer the reads the better, as it improves the specificity of mapping [32,33]. The short-read sequencing technologies are the current dominant technology. However, newer approaches that sequence very long-reads, including the complete transcript, are emerging [34,35]. Although these technologies suffer from higher sequencing error rates and lower quantitative range than short-read technology, they are becoming a powerful tool to correctly annotate full-length transcripts [36,37].

Long- and short-read technologies are different in the sequence yield per run, sequencing accuracy, observed raw error rate, read lengths, insert size and RNA requirement [[37], [38], [39], [40], [41]]. Particularly, read quality is very important for reproducibility and reliability of transcript assembly and quantitation [42]. Short read sequencing quality is commonly assessed by tools such as FastQC (https://www.bioinformatics.babraham.ac.uk/projects/fastqc/). If the quality is poor, tools like Fastx-toolkit (http://hannonlab.cshl.edu/fastx_toolkit/), Trimmomatic [43], PRINSEq. [44], Flexbar [45] and others can be used to trim or filter reads, which can help improve mapping accuracy. The higher sequencing error rate in long-reads requires error correction either by short-reads [39,[46], [47], [48]] or by self-correction of the long-reads [35,49].

## 3 Gene-level and Transcript-level Quantification

At the simplest level of analysis, RNA-Seq data can be considered by mapping it only against a reference transcriptome (not the genome). In transcript-level analyses, all isoforms of a gene are considered separately, whereas in gene-level analyses, all of the isoforms of a gene are merged to form a single unit. For humans and other model organisms, the genome sequences and annotations are relatively complete, particularly for coding genes. Therefore, there is often no need for *de novo* assembly if the research aims are only to assay well annotated genes. However, a choice should be made as to whether the analysis is at the level of the gene or at the transcript. Gene-level analysis is the simplest, and it removes a lot of confounding information related to minor transcript isoforms. In many cases, transcripts have one dominant isoform and several minor isoforms. The measurement of differential expression can often overemphasize changes in minor transcripts whilst the major transcripts are relatively unchanged, making interpretation a challenge [50,51]. However, analysis at the gene-level loses much of the complexity of transcript expression, and is not easily suited to the analysis of particular types of non-coding genes, such as anti-sense or sense intronic transcripts, which are difficult to interpret in gene-level quantification.

In well annotated organisms, gene-level quantification may be all that is required for many purposes. This is because gene-level quantification is less complicated, the properties of genes are relatively well known and the focus is mostly on protein-coding genes. In addition, most highly expressed genes have single dominant isoform [52]. In fact, many studies (*e.g.* [53,54]) skip the assembly of transcripts and only consider well annotated genes from, for example, GENCODE [55]. This has allowed the development of databases that reanalyze very large amounts of data, often from many laboratories, using unified pipelines. For example, Vivian et al. [56] developed the Toil pipeline, to quantitate over 20,000 samples. Other projects include the analysis of cancer samples by the Cancer Genome Atlas (TCGA), involving >8000 samples from >30 cancer and normal cell types [57] and the Genotype Tissue Expression (GTEx) project which has >9000 samples across 53 tissues from 544 healthy individuals [13]. Necsulea et al. [15] used 185 RNA-Seq samples, including previously available and newly generated sequences from six species, to investigate lincRNA (long intergenic non-coding RNA) evolution in tetrapods. Another study [58] used numerous samples to study *in vitro* human cerebral cortex development from human embryonic stem cells. We previously reanalyzed 921 RNA-Seq samples, from 272 mouse tissues and cell types to identify eight major domains of cell type specification [53].

Most pipelines pass through a quantification step. Quantification can be achieved using alignment-based or alignment-free tools. Alignment-based tools align all reads from a sample to a genome or transcriptome, and then using only the mapped reads, count the number of reads that map to an individual transcript or gene. Some of the most common alignment-based tools include RSEM [54], StringTie [59], eXpress [60], TopHat/Cufflinks [61], rQuant [62], MMSEq. [63] and Scallop [64]. These tools have seen widespread use in a range of projects, for example, TopHat/Cufflinks [65], or RSEM [53], the last of which is particularly popular due to its accuracy and user friendly interface. Many quantitative tools are ‘wrappers’ around a lower-level alignment tool which aligns reads to an index of DNA/RNA sequences. Widespread aligners include Bowtie1/2 [66], STAR [67], HISAT1/2 [68], GSNAP [69] or BWA [32]. These tools accept reads and align them to an index, which could be composed of the genome, transcriptome, or any custom index built by the end-user. A list of steps, selected associated tools, and their purposes are presented in Table 1. One advantage of alignment-based quantification methods is their sensitivity [70]. However, this comes with a time and memory cost [[71], [72], [73]], which is due to the requirement to optimally align each read accurately.

**Table 1:** Selected tools for transcript assembly

Process	Tool	Purpose	Input	Output
Read treatment	FastQC	Checks the integrity and quality of reads	Fastq files	Quality charts
	FastX toolkit, Flexbar, Trimommatic	Filters or trims reads	Fastq files	Clean reads; reports
Assembly	Trinity, Trans-ABySS, Oases, SSP, IDBA-tran	Assembles reads without reference	Clean reads	Assembled transcripts
	TOPHAT, STAR, HISAT, HISAT2 with stringTie	Assembles reads with reference annotation	Clean reads, genomic reference, reference annotation	Assembled transcripts
Transcript Classification	BEDtools, glBase	Checks overlap between coordinates	BED, GTF, GFF files	BED, GTF, GFF, report files
	BLAST, BLAT, GMAT, Augustus	Homology based classification	Fatsa files	Alignments, reports
	CPAT, FEELnc, NRC, lncRScan-SVM	Coding potential assessment	GTF or fasta files; reference annotations (mRNA fasta or GTF and genomic fasta)	Coding potential scores, reports
Mapping	TOPHAT, STAR, HISAT, HISAT2, Bowtie, BWA	Aligns reads to transcript or genes	Reads; reference annotations (gtf)	Alignments (bam, sam)
Quantification	RSEM, StringTie, bam-readcount, featureCount	Estimates transcript abundance	Alignment files	Abundance estimates
	Sailfish, Salmon, Kallisto	Estimates abundance without alignment	Reads; reference annotations	Abundance estimates

To speed up quantification methods, alignment-free tools have been developed. Alignment-free quantification strategies use some variant of k-mer counting within the sequencing libraries (*i.e.* counting all the k-mers in a sequencing library, without looking at the genome), which can be collected very fast, rather than align every single read and then quantitate afterwards, as in alignment-based strategies. Sailfish [73] or Salmon [72] count the k-mers and then uses only the unique k-mers to quantify expression. In these approaches, only the final unique k-mers need to be mapped to the transcriptome to identify the transcript, leading to a substantial increase in speed, at the cost of a small loss in sensitivity. A problem with these tools is that they only consider unique k-mers, and so are unsuited to the quantification of repeat-derived RNAs, and they are most suited to transcript-level quantification, as they exploit unique splicing patterns to collect unique k-mers. Indeed, the authors of Kallisto suggest that it is only suited to transcript-level quantification, and gene-level quantification may be misleading [71]. Evaluation of alignment-free methods by Wu et al. [70] revealed that they tend to perform poorly with lowly expressed transcripts or short RNAs. These tools also tend to perform better in well annotated genomes, with good transcript annotations. However, for many users, the loss of some sensitivity is a good tradeoff for large speed improvements. Other related expression quantification tools such as HTSEq. [74] and featureCounts [75] are now increasingly being used. The speed is comparable to those of other alignment-free methods but the sensitivity is improved.

A typical quantification begins after alignment with the number of reads/fragments (or k-mers) that mapped to a transcript or gene. This number depends on the actual expression level, the library size, percent of reads aligning, transcript length, GC content, and other (often hidden) confounding parameters (*e.g.* batch effect, operator bias, *etc.*) [17,76]. To have a better picture of the true expression level, quantification is usually followed by expression normalization. The classic expression unit is Reads Per Kilobase per Million aligned reads (RPKM) [7] or Fragments Per Kilobase transcript per Million aligned (FPKM) [77] which both correct for the library sizes and transcript lengths (RPKM is for single-end reads and FPKM is for paired-end reads). These approaches are conceptually simple, and allow for the comparison of gene expression levels across samples. However, despite their common usage, several studies have pointed out significant flaws in RPKM/FPKM approaches. Highlighted flaws include bias in gene length, GC content and dinucleotide frequency [78], and inconsistency in the averages of the relative molar RNA concentrations across sets of transcripts [79]. Consequently, Transcripts Per Million reads (TPM) was developed as a new unit [79]. However, in our experience we find that the RPKM/FKM or TPM only perform well when the samples under analysis are already closely matched. For example, they came from similar cell types, were sequenced inside the same batch, and do not have much overall variation. When any of these conditions is violated, more robust normalization procedures are required for meaningful quantification. For example, a comparison of normalization methods suggests that RPKM/FPKM approaches were poor in terms of distribution, clustering and false-positive rate, whilst techniques employing mean-normalization of tag counts had superior performance [80]. In our experience, the single most important factor to control for is GC-bias in genes/transcripts, which can help remove batch effects in RNA-Seq samples [19,53], and mean-normalization and related techniques can also remove a lot of confounding problems in RNA-Seq data. One important assumption that the mean-normalization techniques share is that the overall level of RNA is relatively similar between samples, and that the overall variance in gene expression is low. This may not be true in all cases. For example, it has been argued that the overexpression of the oncogene c-*Myc* in tumor cells causes a global amplification of transcriptional output [81]. If the RNA-Seq is mean normalized, this global amplification would be lost as the transcriptional outputs of both samples would be normalized to their means.

## 4 *De novo* Transcript Assembly

For studies that require only gene or transcript-level quantification, robust gene models are required. However, many organisms lack robust gene models, and there is evidence that, even in the extensively studied human genome, the total set of transcripts remains incomplete [82]. This is a particularly acute problem as it is clear that alternative splicing of novel transcripts is a common cell type-specific occurrence. Consequently, in any particular cell type the gene annotations may be incomplete, requiring the assembly of *de novo* transcripts to generate novel biological insight.

Because many of the sequencing technologies involve the fragmentation of transcripts followed by sequencing of relatively short fragments, inferring the original full-length RNA molecules that gave rise to the observed population of short fragments requires accurate reconstruction of a full-length transcript from the assembly of overlapping short fragments. Assembly can be achieved by using the reads alone (*i.e.* without reference to a genome), a useful technique if no genome sequence is available. However, reference-free assembly is less accurate than guided assembly [50,82]. Because many genome sequences are available, several pipelines have been developed to assemble transcripts that take advantage of known genomic features. For example, Pertea et al. [83] proposed a pipeline for transcript assembly using HISAT2 [68] for alignment, followed by StringTie [59] for assembly. Another pipeline [61] used TopHat [84] followed by Cufflinks [77]. Other assemblers include IsoSCM [85] and Scallop [64]. The assembly of short-reads onto longer transcripts is a challenging computational problem that has seen the development of many algorithmic approaches. However, the accurate reconstruction of transcript models remains a problem [82]. For example, in an assessment of 24 protocol variants involving 14 independent computational methods, Steijger et al. [50] reported that the assembly of complete isoform structures was overall poor using short-read RNA-Seq data in the human genome, with many missing exons and incorrect splice junctions. Ultimately, there is no single best pipeline for all cases, and instead there is competition between competing tools and techniques [41,42].

One advantage of *de novo* assembly from short-reads is that it can be used to study gene expression from any species and cell type within a species [[86], [87], [88], [89]]. *De novo* assembly is dependent on the mutual overlap of fragments that can be chained together to infer transcript models. For highly expressed genes with relatively simple transcript models and fewer splicing variants, this may be reliable to a certain extent. However, for lowly expressed genes, genes with complex splicing patterns, *de novo* assembly from short-reads is not reliable [37,50]. The full-length of a gene might not be recovered [90,91]. Hence, the accurate transcription start site might be missed. The accurate detection of the transcription start site (TSS) is important for experimental techniques like CRISPR screens that work best when the sgRNA is targeted within 100–200 bp of the true TSS [92,93]. DeepCAGE technologies have been a powerful addition to the transcript assembly toolbox as they only sequence the TSS [94]. However, this leads to challenges in inferring which transcript the TSS belongs to [95]. Consequently, transcript assembly is best approached with a combination of tools and experimental techniques. Wang and Gribskokv [90] recently reviewed different *de novo* assembly tools and highlighted the strengths and weaknesses of each of the eight tools considered. *De novo* assembly tools include Trans-ABySS [96], Trinity [91], Oases [97], SSP [98], IDBA-tran [99], Rockhopper2 [87] and BinPacker [100]. An important option to consider in *de novo* transcript assembly is the k-mer size. K-mer size is the length of oligonucleotides that the reads are “decomposed” into, to prime assembly. A number of the tools then use de-Bruijin graphs to link the k-mers together and build transcript models [91,99]. Whereas a larger k-mer size improves speed, smaller k-mer size improves sensitivity. The tradeoff between the two may not always be obvious. Ultimately the use of short-read sequences to assemble transcripts can be challenging as the small fragments (typically 300 bp) mean that only small parts of the transcript can be observed, and some guesswork must be made to stitch fragments together. A number of simulation-based benchmarking [101] and spiked-in control [102,103] tools have been developed to optimize RNA-Seq experiments.

Recent studies are taking advantage of long-read technology that can cover intact transcripts, and reveal splice patterns [36,82,[104], [105], [106]]. Long reads generally have higher sequencing error rates and lower yields. However, the technology is now being deployed more widely, either independently [35] or in combination with short-reads [37], to address biological questions. Sharon et al. [35] reported a survey of the human transcriptome using long-read sequences of 20 human samples. Au et al. [37] combined both short and long-reads for isoform identification and quantification to characterize human ESC transcriptome. Abdel-Ghany et al. [107] surveyed sorghum transcriptome with single molecule long-reads. Wu and Ben-Yehezkel [108] used long-reads to survey the transcriptome of three human tissue samples. These studies show that the full-length of many transcripts could be retrieved and reported many previously unannotated transcripts. Likewise, Chen et al. [36] reported a transcriptome atlas of rabbit using both long and short-reads, an important innovation in rabbit, which lacked extensive sequence data to assemble transcripts. The widespread adoption of long-read technology is likely to significantly enhance the accuracy of transcript assembly.

Although long-reads have been reported to perform better than short-reads in transcript assembly [35,37,109], the bioinformatics tools and pipelines are still evolving. For example, Au et al. [37] and Chen et al. [36] combined both short and long-reads for better assembly. In those studies, short-reads with higher sequencing accuracy, were used to correct long-reads. Some of the error-correction tools include LSC [39], LSCplus [46] and loRDEC [48]. The Pacbio company provides the Isoseq3 pipeline (https://github.com/PacificBiosciences/IsoSeq3) that uses long-reads exclusively to get near full-length transcripts, similar to the pipeline of Sharon et al. [35]. A number of tools have been used for aligning long-reads. Au et al. [37] used BLAT [110]. Križanović et al. [40] compared the performance of STAR [111], GMAP [112] and BLASR [113]. Another tool that has been used for aligning long-reads is Minimap2 [114]. Ultimately the use of long-reads for transcript assembly remains work in progress, but shows great promise to improve transcript annotations.

## 5 Detection of Coding and Long Non-coding RNAs From RNA-Seq data

Over the last few years, many non-coding RNAs have been discovered that are increasingly being assigned biological functions [[115], [116], [117], [118]]. However, the detection and annotation of these transcripts is challenging as they are generally lowly expressed, often contain repetitive regions (see below) [119], and even the classification of coding *versus* non-coding is a surprisingly complex problem [[120], [121], [122]] as it is not simply a case of just measuring the longest coding sequence in the transcript. Clamp et al. [122] argued that open reading frames are randomly present in the genome and that their presence is not enough to classify a transcript as coding. Similarly, many genes have multiple isoforms [52,123], and a gene may have both protein-coding and a non-coding transcript, which will confuse sequence homology based searches as the non-coding transcript may contain stretches of truncated coding sequence [124,125]. Indeed, Jungreis et al. [126] argued that nearly all new protein-coding predictions in the CHESS database [127] are not protein-coding.

The first check on the nature of an assembled transcript is to overlap the coordinates with known transcripts. This can be done with tools such as BEDTools [128] and glbase [129]. Homology-based approaches can also indicate the possibility of coding potential, for example, BLAST [130], BLAT [110], GMAP [112], AUGUSTUS [131] and others. These tools classify transcripts based on the similarity of the amino acid sequences of their translated transcripts to known protein-coding genes. Coding potential is thus measured as the similarity of a transcript to other coding transcripts. The obvious limitation is in cases where no related coding sequence is available. Several other tools take a different approach to assess the coding potential of a transcript. These tools use the properties of known coding or non-coding transcripts to test the likelihood that a transcript codes for a protein or not. For example, coding potential can be estimated by machine learning approaches that discriminate transcripts based on combinations of properties such as transcript length, length of open reading frame (ORF), ORF coverage, k-mer frequency, Fickett score or codon usage bias. Several tools, such as CPAT [132], FEELnc [133], lncRScan-SVM [134] and NRC [135], use the same overall approach, but optimize for different techniques or scores. Machine learning approaches rely less on homology and learn the properties of known transcripts to predict coding or non-coding transcripts, making them suitable to annotate novel coding and non-coding genes. These approaches are especially useful in organisms that lack good gene annotations, as demonstrated by the use of FEELnc to annotate coding and non-coding transcripts in the dog genome [133]. Table 2 summarizes the tools available for classifying transcripts into coding and non-coding.

**Table 2:** -Example tools and approaches for classifying coding and non-coding transcripts.

Approach	Instances	Example tools
Coordinate overlap	Known coordinates from good genome annotations	BEDTools, glbase
Homology based	Known sequence and reasonable databases	BLAST, BLAT, GMAP, AUGUSTUS
Machine learning	Characterizing features of coding and noncoding transcripts	CPAT, FELLnc, lncRScan-SVM, NRC

## 6 Assembly of Transposable Elements into Long Non-coding RNAs and Splicing into Coding Genes

Transposable elements (TEs) are the most common type of genomic unit within genomes, outnumbering protein coding exons by a considerable margin [136]. TEs consist of two major types. The first is the DNA transposons that replicate mostly by a cut-and-paste mechanism and rely on the DNA repair mechanism or cell division to replicate. The second major type is the RNA transposons that use an RNA intermediate. The RNA transposons can be further subdivided into the long and short interspersed elements (LINEs and SINEs) and the long-terminal repeats (LTRs), which are endogenous retroviruses [137,138]. TEs have often been considered as parasites, or background genomic noise, but they are increasingly appreciated for their expanding number of roles in genome evolution, and gene regulation rewiring [[139], [140], [141]]. Importantly, TEs are a major contributor to the sequences of non-coding RNAs [119] and several studies have observed the splicing of distal TE exons into coding sequences that did not contain any TEs [[142], [143], [144]]. For example, MERVL expression marks a subpopulation of totipotent cells in cultures of embryonic stem cells [145,146], and a distinctive feature of these cells is the splicing of MERVLs into coding genes, such as *Zfp352* and *Apol7b* [147].

**Fig. 2:**
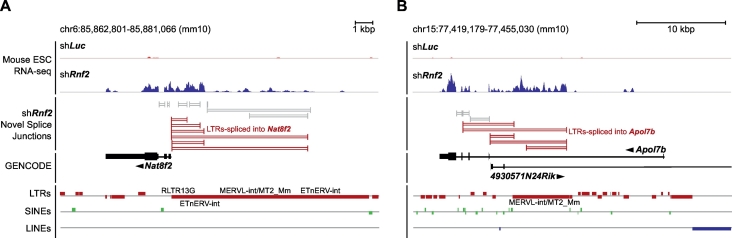
Splicing of transposable elements into genes. RNA-Seq data from mouse ESCs showing the control (sh*Luc*) or a knockdown of the RING finger domain, polycomb 1 protein RNF2 (sh*Rnf2*), that leads to the activation of expression of two genes: *Nat8f2* (panel A) and *Apol7b* (panel B). For each genomic view, the first row shows the short-read RNA-Seq read pileup density in the control (sh*Luc*; red) and knockdown (sh*Rnf2*; blue) experiments. The second row shows the novel splice junctions detected in the RNA-Seq data when *Rnf2* is knocked down (sh*Rnf2*). Splice junctions that join an exon of *Nat8f2* or *Apol7b* to an LTR are indicated in red, other splice junctions are indicated in grey. The third row shows the GENCODE genes at this locus. The fourth row shows the locations of the LTRs (red), SINEs (green) and LINEs (blue). LTRs that show evidence of splicing into *Nat8f2* or *Apol7b* are labeled. Data is from GSE108091 [147]. Reads were aligned to the mm10 genome using STAR [111], with the parameters described in [147]. (For interpretation of the references to colour in this figure legend, the reader is referred to the web version of this article.)

There are significant challenges in the analysis of TEs, and splicing into alternate transcripts. The repetitive nature of TEs means that accurate mapping of the short-reads from typical RNA-Seq protocols is difficult [148]. As most TEs have between several tens to several million copies through the genome [136,137], there remains some uncertainty about where exactly a TE-derived RNA-Seq read is derived from. This can be mitigated, to some extent, by analyzing TEs as ‘metagenes’ and aligning the reads across all genomic copies of the TE and then merging the reads to treat each TE type as a single entity [147,149]. This approach is likely the most robust, until very long-reads become widely available [82]. However, this approach discards the genomic context of the TE copies, which leaves a lot of potential insight unexplored. For example, TE expression can act as sense and anti-sense regulatory RNAs [148,150], and can be spliced into normal coding genes to make chimeric transcripts [117,151]. Consequently, it would be preferable to assemble transcripts that include reads derived from TEs, whilst maintaining their genomic context. Another problem is the relationship between TEs and long non-coding RNAs. The problem can be illustrated by looking at the lincRNA *Trp53cor1* (lincRNA-p21), which has a functional role in somatic cell reprogramming and several other biological processes [152]. *Trp53cor1* transcript contains an L2b LINE, a MLTR14 LTR, and 7 SINEs (2xAlu, 1xB2, 3xB4, and 1xMIR) [136], and because the TEs exist as multiple genomic copies, reads are often multi-mapped to several genomic locations [153]. Similar problems occur when looking for TEs that become spliced into transcripts, although in this case it is possible to look for paired-end reads where one of the pairs is uniquely mapped, whilst the other pair is multi-mapped inside a TE. An example is the splicing of MERVL TEs into coding genes (Fig. 2A, B) [147,154]. Precise mapping of reads to TEs is further challenging as TEs themselves can contain introns [139,[155], [156], [157]], and spliced transcripts can occur in the middle of TEs. This is surely a contributing factor to the problems in assembling transcripts [158].

To date, no systematic analysis of the best practices for the analysis of TE-derived transcripts has been performed. Researchers usually use a host of tools that were originally designed for the assembly of non-TE containing coding genes. It is unclear if these tools are ideal for the task of assembling TE containing transcripts. Attempts have been made for specialized analysis of TEs. For example, the LIONS [159] analysis suite is a wrapper around the cufflinks [77] transcript assembler that focuses on accurately determining the transcriptional initiation start site for the TE. However, the authors caution that the suite is inaccurate for lowly expressed transcripts.

Finally, the assembly of TEs into transcripts is made more complex as the number of TEs, and their precise genomic locations change between different experimental strains of *Arabidopsis* and Mouse [[160], [161], [162]], and also across human populations [163]. Consequently, the reference genomes cannot be considered the ground truth for TEs. Researchers should be careful in any analysis involving TEs that are known to be polymorphic. For example, the MuLV TE family is different between mice lines [162]. Care should be taken with TE types that are still active in the human genome, for example the various subfamilies of Alu, L1 and SVA TEs [[164], [165], [166]]. TEs are nonetheless important regulators and components of long non-coding RNAs and are often found in the UTRs of coding genes, where they may work as regulatory domains for RNAs, something akin to protein regulatory domains [161]. Consequently, it is important to accurately determine the pattern of assembly of TEs into transcripts, and best practices should be explored.

## 7 Available Annotation Resources

Annotations are useful at two stages of transcriptome analyses. First, reference annotations are useful in guiding the assembly tools such as STAR [111], HISAT [68], Tophat [65] and others. Second, annotations are useful in determining the nature of the newly assembled transcripts. The decision of whether a transcript is known or novel depends on its presence, or the presence of its homolog in an annotation database. There are many annotation databases available, for example RefSeq. [167], Ensembl [168] and UCSC [169] databases. These databases collate other databases to form a curated set of data that is often the first port of call for researchers looking for high quality annotations.

GENCODE [158] contains the reference annotations for mouse and human, and efforts are being made for other model organisms such as *Drosophila sp* and *Caenorhabditis elegans*. The choice of the annotation resources to use depends on the species and the tissues being investigated. Additionally, there are specific databases that address certain needs. For example Intropolis [170] is a large-scale dataset of splice junctions in the human genome. Similarly, different annotation databases follow different strategies on inclusion; GENCODE tends to require a higher burden of evidence before calling a gene, whilst other databases contain a much wider set of data with lower requirements. For example, GENCODE reports 16,193 long non-coding RNAs, LNCipedia 56,946 [171], and NONCODE reports 96,308 [172]. Clearly, care needs to be taken by the researcher on which annotation database to use, in human and mouse GENCODE is most suitable, but if the researcher is interested in non-coding transcripts then other more extensive databases may need to be considered.

## 8 Reproducible Sharing of Bioinformatics Pipelines

Reproducibility is a potential problem in genomic research, as tools are often chained together to form a ‘pipeline’, and changes in one step of the pipeline can have downstream effects on subsequent tools. Additionally, researchers often prefer different tools in different steps when trying to optimize analysis for their preferred strategy. To enhance reproducibility, pipelines are often presented as part of a published report. For example, Pertea et al. [83] presented a pipeline for RNA-Seq, and Trapnell et al. [61] presented a pipeline for differential expression. Toil pipeline [56] enables reproducible analyses of big data using tools such as Kallisto [71]. The bioinformatics pipelines used in the ENCODE project are available at their website and are well documented (www.encodeproject.org). These can be a valuable source of example analysis strategies.

In addition, there are computational tools that are specifically aimed at reproducible bioinformatics analysis. Snakemake (https://snakemake.readthedocs.io/en/stable/), Nextflow (https://github.com/nextflow-io) and Docker (https://github.com/ngs-docs/2015-nov-docker/)are different platforms with pipelines for reproducible transcriptome analysis. The tools accept the annotations (genome sequences and gene annotations) and short-reads as input and run specific bioinformatics analyses. SystemPipeR [173] is another tool that provides pre-configured workflows and reporting templates for numerous NGS data including RNA-Seq. Using these platforms, more comprehensive and user-friendly tools have been produced. For example, Visualization Pipeline for RNA-Seq analysis (VIPER) is a user-friendly and comprehensive analysis workflow that uses Snakemake [174]. Another Snakemake-based pipeline, hppRNA [175], is a parameter-free pipeline that can be used for numerous samples. While these tools are user-friendly, convenient and require minimal bioinformatics experience, some specific cases require adjustment of certain parameters that require familiarity with the working of the bioinformatics tools.

## 9 Tools for the Job: RNA-Seq as a Powerful Tool for Gene Quantification

Often time, the decision of which tool is optimum has to be taken at one point or the other. This is sometimes a Herculean decision because of the enormity of the tools available [40,176]. Even experienced bioinformaticians have to make such decisions in the process of optimizing the pipeline. A number of factors determine which tools and pipelines to use (see Table 3). Some of the factors to consider include the purpose of the analyses, the quality of annotation, the type of sequence reads available, available computational resources, nature of the transcripts of interest (transposable elements or non-duplicate genes), the level (gene or transcript level), desired speed of analysis and familiarity with bioinformatics procedure. These factors should be considered before adopting any published bioinformatics tools or pipelines.

**Table 3:** Example tools for different stages of RNA-seq.

Analysis	Conditions	When to use	Recommended read type	Useful tools	Possible pipeline
Mapping	Transcripts as reference	Reliable and near-complete annotations	Short reads	Bowtie2, STAR, HISAT	Trinity package
	Genome sequences as reference	Poor transcript annotation, new assembly	Long reads	GMAP, Minimap2, STAR	
Quantification	Good annotation	Normal expression level estimation	Short read	RSEM, Kallisto, Salmon	
	Poor/no annotation	Assembly follwed by quantification	Long and short reads	Hisat2/StringTie, TopHat/Cufflin	Hisat2/StringTie, TopHat/Cufflinks
	Gene level quantification	Comparing genes	Short reads	RSEM, Kallisto, Salmon	RSEM
	Count of aligned reads	Expression level from alignments	Sort reads	HTSeq, featureCount	
	Transcript level quantification	Interest in isoforms	Short reads	RSEM, StringTie, TopHat	
	Limited computational resources	Quick estimation	Short reads	Kallisto, Salmon	Toil
	Repeatitive element	Transposable element quantification	Long and short reads	RSEM with special parameters	LIONS
Assembly	Good annotation	*De novo* transcript discovery	Long and short reads	Isoseq followed by GMAP, Minimap or STAR	
	Poor/no annotation	Good transcript annotation	Long reads	Isoseq followed by GMAP, Minimap or STAR	
	Repeatitive element	Transposable element expression	Long reads	Isoseq followed by GMAP, Minimap or STAR	
Automated process	Sequential analyses	Limited bioinformatics skills	Long or short reads	Numerous tools	SystemPipeR, VIPER, hppRNA

Gene quantification is a powerful tool that has achieved widespread use. Whilst the quantification of RNA cannot capture all cellular variation, it is nonetheless a powerful exploratory tool to understand cellular dynamics in response to changes in cell type, environmental stimuli or the effects of disease. We have mostly discussed bulk RNA-Seq in this review, but cells are heterogeneous mixtures. Single cell RNA-Seq is revealing more heterogeneity in gene expression than expected [177,178], which is challenging traditional definitions of cell type [179,180]. Overall, expression quantification and particularly RNA-Seq is a powerful technique that has led to major insights into biological processes, and has become a key tool for solving future biomedical problems.
